# Persistent overhydration despite significant fluid reduction limits lung function improvement after a single haemodialysis session in CKD patients: A before and after study using BIA

**DOI:** 10.1016/j.clinsp.2025.100821

**Published:** 2025-11-25

**Authors:** Alexander M. Tungu, Elvis Msaki, Jacktan J. Ruhighira, Ashabilan Ebrahim, Davis Ngarashi, Jacqueline G. Shoo, Fredirick L. Mashili

**Affiliations:** a Department of Physiology, Muhimbili University of Health and Allied Sciences, Upanga, Dar es Salaam, Tanzania; b Department of Physiology, Military College of Medical Sciences, Lugalo, Dar es Salaam, Tanzania; c Department of Medical Physiology, University of Dodoma, Dodoma, Tanzania; d Department of Internal Medicine, Muhimbili National Hospital, Upanga, Dar es Salaam, Tanzania; e Research Centre for Health through Physical Activity, Lifestyle, and Sports (HPALS), Division of Physiological Sciences, Department of Human Biology, University of Cape Town, Cape Town, South Africa

**Keywords:** Lung function, Chronic kidney disease, CKD, Spirometer, Haemodialysis, Overhydration, BIA, BIVA

## Abstract

**Background:**

Hemodialysis (HD) is frequently employed to remove excess fluids and potentially enhance lung function in patients with Chronic Kidney Disease (CKD) by alleviating congestion. The authors utilized a combination of traditional Bioelectric Impedance Analysis (BIA) parameters and Bioelectric Impedance Vector Analysis (BIVA) to offer more detailed insights into hydration status before and after a single HD session, and correlated these findings with spirometry.

**Methodology:**

The authors conducted an observational before-and-after dialysis study of 45 clinically stable CKD patients attending routine dialysis care. They assessed fluid status and lung function before and after a four-hour dialysis session using a mulifrequency BIA deviceand a standard spirometer.

**Results:**

Most study participants (71 %, *n* = 32) were overhydrated before hemodialysis. The HD session significantly reduced mean Total Body Water (TBW) [t(44) = -11, *p* < 0.001], Extracellular Water (ECW) [t(44) = -13, *p* < 0.001], Intracellular Water (ICW) [t(44) = -11, *p* < 0.001], and Transcellular Water (TSW) [t(44) = -9, *p* < 0.001]. Despite these reductions, 66 % of participants (*n* = 21) remained overhydrated based on BIVA. Regarding lung function, 73 % of participants (*n* = 33) exhibited abnormal pre-hemodialysis spirometry patterns, with 20 % portraying obstructive, 38 % restrictive, and 16 % mixed patterns. Post-hemodialysis, no significant changes were observed in spirometry parameters or their respective % predicted values and lung function patterns. Interestingly, a significant association was found between spirometry patterns and BIVA-determined hydration status both before and after 4 hours of hemodialysis (χ^2^ = 20, *p* = 0.002).

**Conclusions:**

The 4-hour dialysis session reduced fluid volumes but did not normalize hydration status for most CKD patients and did not improve lung function post-dialysis.

## Highlights

• Severe overhydration often follows long dialysis-free periods in CKD patients.
• Hemodialysis removes excess fluids, potentially reducing lung congestion.
• A single dialysis session reduces fluids but fails to restore normal hydration.
• Persistent overhydration after dialysis limits lung function improvement.
• BIA-guided dialysis monitoring may optimize fluid balance and patient outcomes.

## Introduction

Chronic Kidney Disease (CKD) is a significant global public health challenge with rising prevalence over the past decade.^1^ CKD affects about 13.4 % of the global population, with similar rates in Sub-Saharan Africa (13.9 %) and Tanzania (13.6 %).^2^ CKD is categorized into five stages based on the degree of reduction in the Glomerular Filtration Rate (GFR), with a GFR < 60 mL/min per 1.73 m^2^ for three months or more defining the initial stage.^3^, ^4^, ^5^ The fifth stage, End-Stage Renal Disease (ESRD), is characterized by fluid overload, primarily within the extracellular compartment, which can lead to pulmonary edema and impaired lung function.^6^^,^^7^ These pulmonary complications significantly increase the risk of mortality among ESRD patients.^7^

Maintaining fluid and electrolyte balance in CKD patients requires Renal Replacement Therapy (RRT), including Hemodialysis (HD), peritoneal dialysis, and kidney transplantation.^8^ HD, one of the most widely used RRT methods, removes excess fluids, potentially improving lung function by reducing pulmonary congestion.^9^ However, despite its theoretical benefits, studies on the effects of fluid removal during a single HD session on lung function remain limited, inconsistent, and vary in methodology.^10^, ^11^, ^12^, ^13^, ^14^ Some studies have reported improvements in spirometry parameters ‒ such as Forced Vital Capacity (FVC), Forced Expiratory Volume in the first second (FEV1), FEV1/FVC ratio, Peak Expiratory Flow Rate (PEFR), and Forced Expiratory Flow (FEF 25‒75 %) ‒ after a single HD session, attributing these changes to fluid removal from the lungs.^11^^,^^12^ In contrast, other studies have noted improvements only in men, highlighting potential gender differences in response to HD.^13^^,^^14^ Furthermore, findings across various populations of CKD patients on HD are inconsistent, underscoring the need for further research.^10^

Bioelectrical Impedance Analysis (BIA) provides a non-invasive and rapid technique for assessing hydration status and body fluid compartments, including the Total Body Water (TBW).^1^^,^^8^ BIA offers advantages of safety, ease of use, and cost-effectiveness, particularly in resource-limited settings. When Resistance (R) and reactance (Xc) are normalized for height and plotted, they form a vector, interpreted through Bioelectrical Impedance Vector Analysis (BIVA). Unlike traditional BIA, BIVA uses raw data without predictive equations, offering a real-time, visual tool to distinguish fluid shifts from changes in cell mass. Evidence from Hemodialysis (HD) cohorts ‒ including a South African study, found BIA superior to staff clinical estimates for fluid assessment among CKD patients,^15^confirm its utility in monitoring fluid dynamics during dialysis. Integrating BIVA with standard BIA-derived indices (e.g., TBW, extracellular water, phase angle) therefore provides a more inclusive picture of a patient’s fluid dynamics than either method alone. This simultaneous BIA/BIVA approach is a practical, low-cost alternative to sophisticated imaging or biomarker panels that are often inaccessible in low-resource countries such as Tanzania, and it holds considerable promise for routine HD monitoring where rapid, bedside decisions are critical.

With the growing burden of CKD and the increasing reliance on RRT, understanding the relationship between fluid dynamics, hydration status, and clinical outcomes such as lung function is crucial. Considering that CKD's impact on the lungs is partly due to fluid overload, exploring the association between body fluid changes and spirometry parameters is essential. The 2023 Kidney Disease: Improving Global Outcomes (KDIGO) Clinical Practice Guideline for Hemodialysis Adequacy emphasizes individualized fluid management strategies that integrate both clinical judgment and objective assessment tools, such as BIA, to prevent chronic overhydration and its associated cardiovascular and pulmonary consequences.^16^ This is clinically significant, especially in the first weekly sessions, during which major morbidity and mortality events have been observed to be higher.^10^^,^^17^ This study aimed to document changes in body fluid and hydration status following a single HD session and determine the influence of these changes on lung function among Tanzanian CKD patients at a national hospital in Dar es Salaam, Tanzania.

## Materials and methods

### Study design and setting

The authors conducted a hospital-based, before-and-after observational study among CKD patients at a tertiary referral hospital in Dar es Salaam, Tanzania, from April 19 to June 18, 2021. The hospital serves as a referral center for patients from regional hospitals nationwide and operates 55 dialysis machines across two sites. This study did not affect or interfere with conventional dialysis scheduling, dialysis adequacy determination, or evaluation procedures. All dialysis procedures adhered to Muhimbili Hospital guidelines and clinical protocols. The present work was limited to performing BIA, including BIVA, and spirometry prior to and following the HD session, with no influence on patient management or therapy delivery.

### Study population and participants

The study involved Chronic Kidney Disease (CKD) patients undergoing maintenance Hemodialysis (HD), who were identified and assessed for eligibility through review of patient files and clinical evaluation. Both demographic and clinical characteristics were systematically recorded using a standardized checklist.

The authors consecutively recruited 69 patients attending their first weekly HD session. Eligible participants were clinically stable adults (aged ≥ 18 years) receiving at least twice-weekly HD for a minimum of six months. Patients were excluded if they had Acute Kidney Injury (AKI), a current or past history of smoking, known acute or chronic pulmonary disorders, previous Pulmonary Tuberculosis (PTB), musculoskeletal abnormalities, decompensated heart failure, cardiac arrhythmias, liver cirrhosis, or were unable to perform pulmonary function testing.

**Fig. 2:**
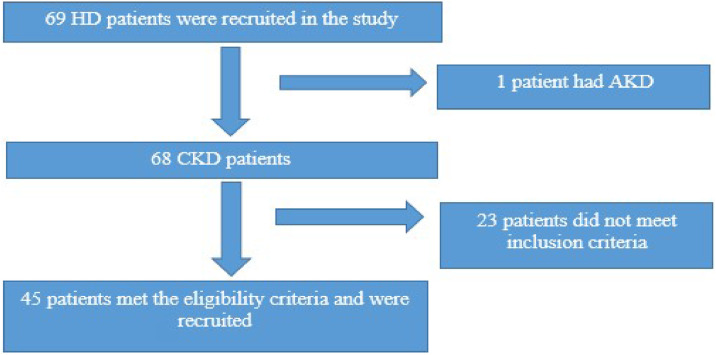
Recruitment procedure.

Following eligibility assessment, 24 patients were excluded based on these criteria, resulting in a final sample size of 45 participants who completed all study procedures (Fig. 2). All participants were enrolled on their first weekly session to minimize the effect of session variability, as HD patients are likely be mostly overhydrated following the weekend interdialytic period.^19^ Sample size estimation was based on a previous study that reported a mean difference and standard deviation in FVC % of 1. Assuming a 15 % nonresponse and a significance level of 0.05, about 45 participants were sufficient to achieve 80 % power to detect a change in FVC %.

### Spirometry assessment

The authors performed spirometry using a Computerized Easy One® spirometer (ndd Medical Technologies, Zurich, Switzerland) before and after four hours of HD. The measured Forced Expiratory Volume in one second (FEV1), Forced Vital Capacity (FVC), Peak Expiratory Flow (PEF), FEV1/FVC ratio, and Forced Expiratory Flow (FEF 25‒75 %) with their respective percentage predicted values were measured before and after a single HD session. Predicted values were obtained from African reference values derived from Global Lung Initiative equations.^20^ Measurements were performed following the American Thoracic Society/European Respiratory Society (ATS/ERS) acceptability and repeatability criteria.^21^ The maneuver was performed in a sitting position to avoid light-headedness, without a nose clip. Patients were instructed to take as deep a breath as possible and blow out as hard and as fast as possible. Furthermore, patients were encouraged, particularly near the end of the maneuver, to keep blowing until no more air came out. The maneuver was performed three times, and the best values were chosen and recorded automatically. All tests were performed by one operator. Two spirograms were discarded due to poor quality, leaving a total of 43 spirograms for final analysis.

### Body fluid assessment

A Multifrequency Quadscan 4000 analyzer (Bodystat, Isle of Man, UK) was used to assess Total Body Water (TBW), Extracellular Water (ECW), Intracellular Water (ICW), and Third Space Water (TSW) using BIA.^22^ Patients were asked to lie on their backs, then inner sensing electrodes were attached to the dorsal surface of the participant’s wrist, and an outer source electrode was placed on the dorsal surface of the third metacarpal bone. The second pair of electrodes was positioned on the ipsilateral leg on the anterior surface of the ankle and the third metatarsal bone, respectively. The electrodes were left in place during the HD session for post-HD tests as recommended by a previous study.^23^

**Fig. 3:**
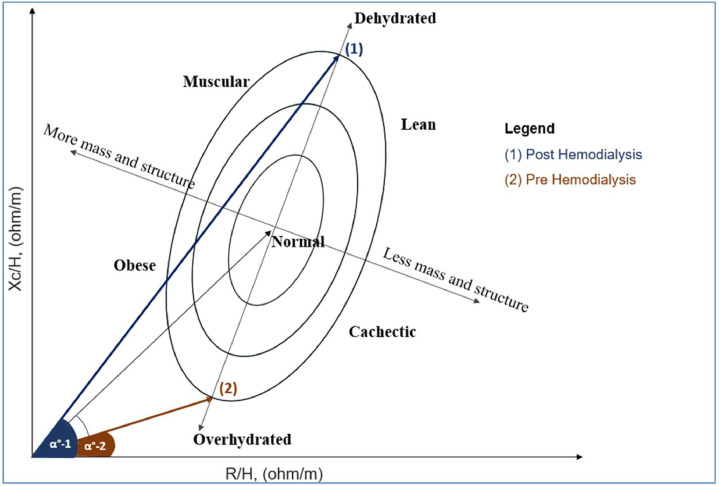
Schematic representation of the ideal Bioelectric Impedance Vector Analysis (BIVA) before and after dialysis, assuming sufficient fluid removal. This figure represents the variations of PhA and vector length concerning dialysis: Both PhA values (α°) (direction) and vector length (magnitude) increase as the participant loses fluid from a state of overhydration (within the overhydration quadrant) to normal hydration (in the middle) and eventually to a state of dehydration (at the far upper quadrant).

Hydration status was assessed by Bioelectrical Impedance Vector Analysis (BIVA), which measures hydration status based on reactance (Xc) and Resistance (R) normalized to height (Fig. 3). The vector position on the RXc graph was interpreted using the 50 %, 75 %, and 95 % tolerance ellipses calculated from a healthy reference population. Individuals whose vectors fell below the lower pole of the 75 % tolerance ellipse were classified as overhydrated, those within the 50 % ellipse as normally hydrated, and those above the upper pole of the 75 % ellipse as dehydrated.^24^^,^^25^

### Statistical analysis

Descriptive statistics were summarized as Arithmetic Mean (AM) with Standard Deviation (SD), and frequencies (n) with %, for continuous and categorical variables, respectively. The mean change in TBW, ECW, ICW, TSW, phase angle, vector length, FEV_1_, FVC, FEV1/FVC ratio, FEF25‒75 %, PEFR, and their respective percentage predicted values were calculated as the difference in post- and pre-HD values (post-HD minus pre-HD). A paired Student *t*-test was used to determine the statistical significance of the mean change in parameters after a single HD session. One-way Analysis of Variance (ANOVA) with the post-hoc Bonferroni test was used to compare mean changes between variables with more than two groups. A linear regression model was used to determine the association between mean changes in body fluid and the changes in lung function parameters after the session while adjusting for age groups, sex, height, predisposing factors, duration of HD, and vascular access. The Fisher-Freeman-Halton exact test was used to assess the representativeness of the sub-analysis group, and the association between spirometry patterns and hydration status.

A subgroup analysis (*n* = 11) was then performed to determine whether a single HD session was adequate by calculating the Urea Reduction Ratio (URR). This subgroup was conveniently selected for the measurement of BUN. The URR was obtained as the ratio of the mean difference in pre- and post-HD BUN to pre-HD BUN (pre-HD BUN minus post-HD BUN/pre-HD BUN). Patients with a ratio of ≥ 65 % were considered adequately hemodialyzed, and vice versa. Data analysis was performed using the Statistical Package for Social Sciences (SPSS) Version 25 at a statistical significance of < 0.05.

### Ethical considerations

This study was conducted in strict adherence to international and national ethical guidelines, including the Declaration of Helsinki, Good Clinical Practice (GCP), and Data Protection Laws (DPL). Ethical approval was obtained from the Institutional Review Board of Muhimbili University of Health and Allied Sciences (MUHAS) with Ethics Committee study protocol number MUHAS-REC-03–2021–534 available at https://irb.muhas.ac.tz/storage/Certificates/Certificate%20-%20472.pdf. Additionally, permission to conduct the study was secured from Muhimbili National Hospital, ensuring institutional compliance. The study is reported following the STROBE Statement.

The study was primarily observational; no additional interventions or procedures beyond routine clinical assessments were introduced. BIA, including BIVA, and spirometry were performed per established clinical guidelines. While BIA and BIVA remain underutilized in Tanzania, it is considered non-invasive and safe methods for assessing hydration status. Spirometry was performed only when clinically indicated, ensuring that participants were not subjected to unnecessary procedures.

Patient safety and confidentiality were strictly maintained. All participants provided written informed consent after being thoroughly briefed on the study's objectives, procedures, and their right to withdraw at any stage without consequences. To protect privacy, each participant was assigned a unique identification number, and all data was stored securely and accessible only to the principal investigators.

Given the vulnerable nature of patients with chronic kidney disease, particular attention was paid to ensuring participant well-being throughout the study. No procedure posed additional risk beyond standard clinical care, and the study was conducted in a manner that prioritized patient safety, dignity, and ethical integrity.

## Results

### Characteristics of the study participants

Participants (*n* = 45) had a mean age of 47 ± 4 years (Table 1). Most participants were male (69 %), and 42 % were overweight or obese. Hypertension was the most common comorbidity factor (95 %). The majority had CKD for 0.5–2 years (58 %), underwent hemodialysis three times weekly (53 %), and maintained fluid intake ≤ 1 L daily (64 %). A subgroup (*n* = 11) analysis of Urea Reduction Ratio (URR) indicated that 83 % achieved adequate hemodialysis after a single hemodialysis session.

**Table 1:** Characteristics of study participants (*n* = 45).

Variable	Parameter	Overall n ( %)	Sub-analysis n ( %)	p-value
Mean age	AM ± SD	47 ± 14	54 ± 10	
Height (cm)	AM ± SD	166 ± 8.5	165 ± 7	
Age groups	18 ‒ 35	10 (22.2)	0 (0.0)	0.355
	36 ‒ 45	11 (24.4)	3 (27.3)	
	46 ‒ 60	16 (35.6)	5 (45.5)	
	> 60	8 (17.8)	3 (27.3)	
Pre-HD BMI (Kg/m^2^)	Underweight (< 18.5)	0 (0.0)	0 (0.0)	1.000
	Normal (18.5‒24.9)	26 (57.8)	6 (54.5)	
	Overweight (25.0‒29.9)	12 (26.7)	3 (27.3)	
	Obese (≥ 30)	7 (15.6)	2 (18.2)	
Post HD BMI (Kg/m^2^)	Underweight (< 18.5)	5 (11.1)	0 (0.0)	0.683
	Normal (18.5‒24.9)	22 (48.9)	6 (54.5)	
	Overweight (25.0‒29.9)	14 (31.1)	3 (27.3)	
	Obese (≥ 30)	4 (15.6)	2 (18.2)	
Sex	Male	31 (68.9)	7 (63.6)	0.732
	Female	14 (31.1)	4 (36.4)	
Comorbidity Factors	Hypertension	33 (73.3)	10 (90.9)	0.631
	Hypertension and Diabetes Mellitus	10 (22.2)	1 (9.1)	
	Malaria	2 (4.4)	0 (0.0)	
Duration of CKD	6 months – 1 year	16 (35.6)	5 (45.5)	0.938
	1 – 2 Years	10 (22.2)	3 (27.3)	
	2 – 3 Years	7 (15.6)	1 (9.1)	
	3 – 5 Years	8 (17.8)	2 (18.2)	
	> 5 Years	4 (8.9)	0 (0.0)	
HD sessions per week	2 times	21 (46.7)	4 (36.4)	0.737
	3 times	24 (53.3)	7 (63.6)	
Vascular Access	Arteriovenous Fistula (AVF)	18 (40.0)	2 (18)	0.428
	Temporary Central Venous Catheter	9 (20.0)	3 (27.3)	
	Permanent Central Venous Catheter	18 (40.0)	6 (54.5)	
Fluid intake per day	≤ 1 Litre	29 (64.4)	6 (54.5)	0.730
	> 1 Litre	16 (35.6)	5 (45.5)	
Adequacy of HD (URR)	Adequate (≥ 65 %)		9 (82.8)	
	Inadequate (< 65 %)		2 (18.2)	

AM±SD, Arithmetic Mean ± Standard Deviation; HD, Haemodialysis; BMI, Body Mass Index, CKD, Chronic Kidney Disease; URR, Urea Ratio.

### Body fluid status

Most participants (71 %, *n* = 32) were overhydrated pre-hemodialysis with 43.8 ± 7 liters TBW, 19.4 ± 2.6 ECW, 22.2 ± 3.3 ICW, and 2.3 ± 1.9 TSW (Table 2). There was a significant reduction in TBW [t(44) = −11, *p* < 0.001], ECW [t(44) = −13, *p* < 0.001], ICW [t(44) = −11, *p* < 0.001], and TSW [t(44) = −9, *p* < 0.001] post-haemodialysis. There was a significant increase in phase angle from 4.1 to 4.6 ohm/m, [t(44) = 8, *p* < 0.001] and vector length from 254 to 291 ohm/m, [t(44) = 12, *p* < 0.001] post-hemodialysis. Despite the decrease in body fluid compartments, most participants (66 %, *n* = 21) remained overhydrated post-dialysis. Similar findings were observed in the subgroup analysis (*n* = 11) except for phase angle.

**Table 2:** Comparison between pre- and post-haemodialysis body fluid, BUN, and spirometry parameters.

Parameter	Pre-HD^a^	Post-HD	*t* (df)	p-value^b^
	Mean ± SD	Mean ± SD		
Total body water (L)	43.82 ± 6.75	40.24 ± 6.79	−11.2 (44)	<0.001
Extracellular water (L)	19.42 ± 2.55	17.77 ± 2.37	−12.7 (44)	<0.001
Intracellular water (L)	22.22 ± 3.25	20.94 ± 3.16	−11.3 (44)	<0.001
Third Space Water (L)	2.26 ± 1.90	1.55 ± 1.74	−8.6 (44)	<0.001
Phase Angle (0)	4.07 ± 1.13	4.57 ± 1.43	7.0 (44)	<0.001
Vector Length (Ohm/M)	253.9 ± 47.8	290.7 ± 58.5	11.7 (44)	<0.001
BUN (mg/dL, *n* = 11)**^§^**	18.5 ± 8.2	5.45 ± 3.15	−7 (10)	<0.001
FEV1 (L)	1.83 ± 0.68	1.84 ± 0.66	−0.1 (44)	0.909
FEV1 %	65.86 ± 20.8	66.44 ± 19.9	0.2 (44)	0.746
FVC (L)	2.46 ± 0.95	2.50 ± 0.86	−0.7 (44)	0.528
FVC %	70.7 ± 21.2	71.9 ± 19.3	−0.5 (44)	0.548
FEV1/FVC	0.75 ± 0.09	0.74 ± 0.12	0.2 (44)	0.588
PEFR (L/min)	309 ± 122	316 ± 124	1.1 (44)	0.503
PEFR %	69.1 ± 22.8	71.0 ± 23.2	0.6 (44)	0.499
FEF25‒75 % (L/s)	1.56 ± 1.0	1.54 ± 0.81	0.3 (44)	0.895
FEF25‒75 %	53.3 ± 28.0	55.5 ± 28.6	0.2 (44)	0.587

a Haemodialysis,.

b Only p-values > 0.016 were considered significant after adjusting for multiple comparisons by Bonferroni correction.

The reduction in fluid volume in all four body compartments was greater among those aged 60 years and above compared to those in the 18‒35, 36‒45, and 46‒60 age groups (ANOVA with post-hoc Bonferroni test, *p* < 0.01, respectively). In addition, the permanent central venous catheter was associated with a significant reduction in TSW compared to the temporary central venous catheter and arterial venous fistula. However, fluid intake, hemodialysis sessions per week, height, predisposing factors, and sex had no significant effect on the mean change in body fluid in any of the four compartments.

### Spirometry parameters

**Fig. 4:**
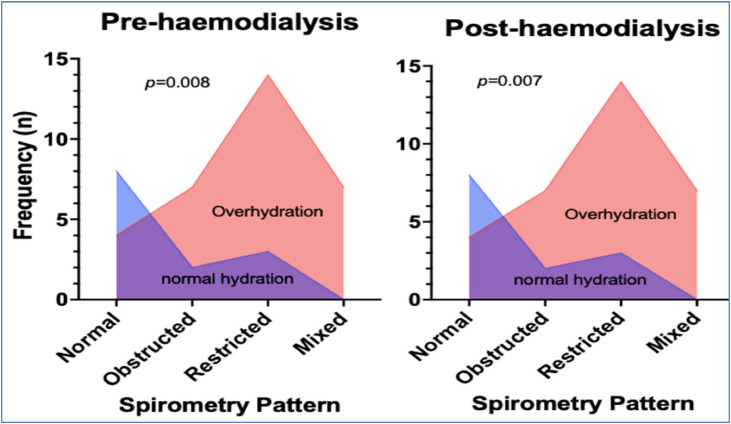
Distribution of lung function patterns (normal, obstructive, restrictive, and mixed) by hydration status before and after haemodialysis. The majority of participants were overhydrated (red area) compared to the smaller proportion with normal hydration (blue area) before haemodialysis and associated with spirometry pattern (*p* = 0.008). Despite significant fluid removal during haemodialysis, the hydration status remained largely unchanged, with a persistent predominance of overhydration compared to normal hydration also associated with spirometry pattern (*p* = 0.007).

The FEV_1_, FVC, FEV1/FVC ratio, FEF25‒75 %, PEFR, and their respective predicted percentage values pre- and post-hemodialysis are presented in Table 2. The majority (73 %, *n* = 33) had abnormal spirometry lung function patterns pre-hemodialysis, whereby 20 % had an obstructed pattern, 38 % had restricted, and 16 % had mixed. The authors found no statistically significant changes in all spirometry parameters post-hemodialysis, even after adjusting for age, vascular access method, height, sex, predisposing factors, and duration of hemodialysis. The spirometry lung function patterns remained relatively unchanged post-hemodialysis. Spirometry patterns were associated with hydration status both pre- and post-hemodialysis (Fig. 4). Likewise, the authors found no statistically significant changes in all spirometry parameters post-hemodialysis in the subgroup analysis (*n* = 11).

## Discussion

The present study revealed significant reductions in all body fluid compartments following a single hemodialysis session. However, BIVA showed that a substantial proportion of participants remained overhydrated. Despite fluid reduction in individual body fluid compartments, the authors observed no significant improvement in post-dialysis lung function parameters. These findings underscore the challenges faced in managing fluid overload in Chronic Kidney Disease (CKD) patients, highlighting the limitations of a single hemodialysis session in achieving optimal hydration and lung function improvement, and calling for the use of technology in determining and monitoring fluid reduction during dialysis.

Consistent with previous reports, 71 % of participants in the present study were overhydrated before dialysis.^11^ The 4-hour hemodialysis session decreased Total Body Water (TBW) and its compartments, aligning with findings by Tangvoraphonkchai et al.^26^ However, unlike their study, which noted no significant change in the Intracellular Compartment (ICW), the authors observed a reduction in ICW, possibly due to fluid migration dynamics during dialysis. A study in Korea similarly indicated that while ECW was predominantly reduced initially, the mean % reduction between ECW and ICW did not differ significantly by the end of the session.^27^ The discrepancies in findings may be attributed to variations in dialysis duration and patient characteristics.

The use of Bioelectric Impedance Vector Analysis (BIVA), one of the unique attributes of the present study, provided a comprehensive hydration assessment, showing that many participants remained overhydrated post-dialysis. Most individuals classified as severely overhydrated before treatment retained this status afterward. This suggests that while hemodialysis effectively reduces fluid volumes, achieving normal hydration levels within a single session remains challenging. In the current study, patient recruitment and measurements were consistently conducted on the first dialysis day of the week, typically following the longest interdialytic interval when fluid accumulation was most pronounced.^19^ This timing highlights that, beyond insufficient fluid removal during a single session, inadequate overall fluid management may contribute to persistent overhydration post-hemodialysis. This finding suggests variability in treatment response, influenced by factors such as baseline fluid status, comorbid conditions, dietary and fluid intake habits, and ultrafiltration rates.^19^^,^^28^

Moreover, HD adequacy was measured by Urea Reduction Ratio (URR), which does not account for fluid removal.^29^ While the present study demonstrated that most participants achieved a URR of ≥ 65 %, indicating adequate waste removal during HD, the persistent overhydration observed in many patients remains a concern. This discrepancy suggests that, while waste extraction was clinically adequate, management of fluid dynamics was less optimal. This finding emphasizes the need for monitored dialysis interventions that not only focus on solute clearance but also prioritize hydration status assessment and management, ensuring comprehensive care for CKD patients.^16^^,^^17^ It is important to note, however, that URR was assessed in only 11 of the 45 patients, and the observed adequacy may reflect limited statistical power within this small subgroup.

Integrating BIVA in the present study is particularly interesting, as it represents a promising method for classifying hydration and body adiposity status.^30^ Previous studies using BIVA have demonstrated its effectiveness in distinguishing between hydration states, making it a valuable tool for assessing CKD patients.^30^^,^^31^ Incorporating BIVA alongside other fluid volume measurements from Bioelectrical Impedance Analysis (BIA) enhances the monitoring of fluid dynamics and hydration status during hemodialysis.^30^^,^^32^^,^^33^ Collectively, these findings and others highlight the complexity of fluid management in CKD patients, emphasizing the need for tailored dialysis interventions that leverage technology and effective algorithms, such as BIVA, to address the unique hydration profiles of each patient and guide appropriate fluid reduction. The integration may present a non-invasive, low-cost alternative to complex methods or biomarkers,^34^ which may not be easily accessible in resource-limited settings.^30^^,^^31^

Despite significant reductions in fluid volumes, the authors did not observe statistically significant changes in lung function parameters after hemodialysis. Anees et al. reported similar findings and found no significant differences in FEV1, FVC, and FEV1/FVC ratios pre- and post-dialysis^35^. Conversely, other studies found significant improvements in FEV1 and FVC following hemodialysis^36^, ^37^. The variability in results across studies may stem from differences in patient demographics, kidney disease severity, and the adequacy of fluid removal during dialysis. Nevertheless, a consistent observation is that there is an association between hydration status and spirometry patterns.

Additionally, the authors observed a close relationship between hydration status and spirometry patterns, which may explain the lack of improvement in lung function despite fluid reductions. The persistent defective spirometry pattern, while partly explained by persistence of overhydration, may indicate underlying pulmonary limitations independent of fluid status. The consistently below-normal spirometry values^38^ suggest that patients, despite the absence of reported respiratory illnesses, may have undiagnosed respiratory conditions causing persistent dysfunction regardless of hemodialysis effects. Therefore, the potential for quantifiable spirometric improvement or reversibility may have been limited by unidentified or subclinical pre-existing respiratory pathologies.^39^ Pulmonary congestion in the interstitial and peribronchial spaces may persist beyond a single dialysis session, thereby maintaining respiratory dysfunction.^40^ Additionally, atelectasis due to prolonged overhydration and reduced mobility could limit expected spirometric improvement.^41^ Another factor for the lack of spirometric improvement could be restrictive lung mechanics, possibly due to uremic effects, diaphragm dysfunctions, or chronic inflammatory effects.^39^ These factors could independently or synergistically limit the reversibility of pulmonary impairment even when volume status is normalized.

**Fig. 1:**
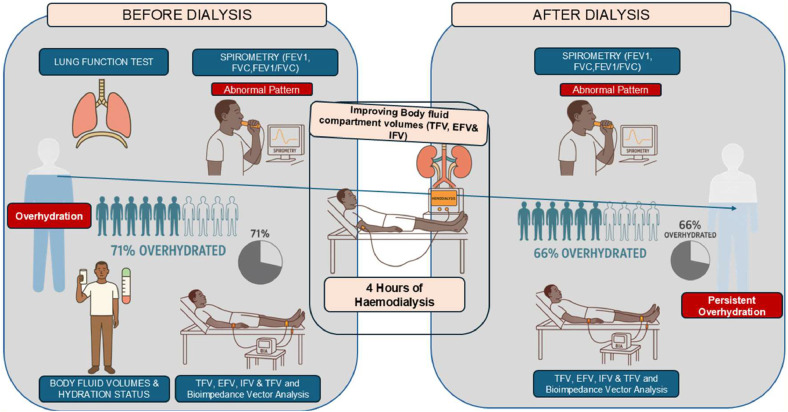
Visual abstract.

In conclusion, the present findings indicate that persistent overhydration, despite significant fluid reduction in individual body fluid compartments, limits lung function improvement after a single haemodialysis session in CKD patients. These results highlight the need for more individualized fluid management strategies accounting for each patient’s unique hydration needs. The integration of accessible and user-friendly technologies such as Bioelectrical Impedance Analysis (BIA), combined with multiple assessment metrics, including Bioelectrical Impedance Vector Analysis (BIVA) and fluid compartment dynamics, can play a crucial role in guiding optimal fluid reduction strategies and improving patient outcomes. Future studies with larger sample sizes are needed to establish specific thresholds for clinically meaningful fluid reductions to better inform dialysis practices, particularly in resource-limited settings (Fig. 1).

### Strengths and limitations

To the best of our knowledge, this is the first study utilizing multiple metrics of the BIA technology in monitoring fluid volume dynamics and hydration status in patients on maintenance HD in Tanzania. These findings will, therefore, stimulate its wider use within the country and beyond. A key strength of this study was the combination of multiple BIA parameters to triangulate results and interpretation, which provided detailed assessments of body fluid compartments and hydration status. The tight follow-up of patients during dialysis allowed for accurate tracking of changes. The present study adds valuable data to the body of evidence in this important yet under-researched area.

This study used consecutive sampling from a single dialysis center without altering routine clinical schedules, which limited control over recruitment timing and the availability of certain measurements, such as BUN and URR. For similar reasons, the sample size was also limited. While this approach preserved real-world clinical practice, it may have introduced selection bias and limited the completeness of data, thus warranting cautious interpretation of the findings. Moreover, while the exclusion criteria tried to exclude those with known respiratory pathologies, the authors did not account for undiagnosed or residual pulmonary conditions, such as subclinical fibrosis or restrictive lung defects, which may have influenced spirometry results.

## Abbreviations

BIA, Bioelectrical Impedance Analysis; BIVA, Bioelectrical Impedance Vector Analysis; CKD, Chronic Kidney Disease; ECW, Extracellular Water; ESRD, End-Stage Renal Disease; FEV1, Forced Expiratory Volume in the first second; FVC, Forced Vital Capacity; ICW, Intracellular Water; KDIGO, Kidney Disease: Improving Global Outcomes; PEFR, Peak Expiratory Flow Rate; RRT, Renal Replacement Therapy; TBW, Total Body Water.

## Funding

The bioelectric impedance analysis device was purchased with funds from GlaxoSmithKline R&D (Africa NCD Open Lab grant number 3000,034,576). The funder was not involved in any way during data collection or any activity directly related to the execution and reporting of the study*.*

## Authors’ contributions

E.M. contributed to the conceptualization and design of the study, data collection, data analysis, and manuscript writing. J.G.S. participated in patient recruitment, data collection, and manuscript writing. J.J.R. contributed to the conceptualization and was involved in data analysis and manuscript writing. A.E. and D.N. assisted in drafting and finalizing the manuscript. A.M.T. and F.L.M. contributed to the conceptualization, supervision of study execution, data analysis, manuscript writing, and finalization. All authors reviewed and approved the final manuscript.

## Ethics approval and consent to participate

The study adhered to international and national ethical guidelines, including the Declaration of Helsinki, Good Clinical Practice (GCP), and Data Protection Laws (DPL). Ethical approval was obtained from the Institutional Review Board of Muhimbili University of Health and Allied Sciences with Ethics Committee study protocol number MUHAS-REC-03–2021–534 available at https://irb.muhas.ac.tz/storage/Certificates/Certificate%20-%20472.pdf. Permission to conduct the study was granted by the Teaching, Research, and Consultancy Unit at Muhimbili National Hospital with reference number MNH/TRCU/Perm/2021/098).

Since the study was observational, no additional interventions beyond routine clinical assessments were introduced. Bioelectrical Impedance Analysis (BIA) and spirometry ‒ both widely used in dialysis patients ‒ were performed following established clinical guidelines, ensuring patient safety and adherence to standard care. Written informed consent was obtained from all participants, who were fully briefed on the study's purpose, procedures, and their right to withdraw at any time without consequence. Confidentiality was maintained by assigning unique identification codes and securing all data, accessible only to the principal investigators.

## Consent for publication

Not applicable.

## Data availability

The data presented in this study are available upon request from the corresponding author.

## Declaration of competing interest

The authors declare no conflicts of interest.
